# Do hereditary syndrome-related gynecologic cancers have any specific features?

**DOI:** 10.1007/s13244-015-0425-x

**Published:** 2015-09-04

**Authors:** Nelson Neto, Teresa Margarida Cunha

**Affiliations:** Radiology Department, Centro Hospitalar de Lisboa Ocidental, Estrada do Forte do Alto do Duque, 1449-005 Lisboa, Portugal; Radiology Department, Instituto Português de Oncologia de Lisboa Francisco Gentil, Rua Professor Lima Basto, 1009-023 Lisboa, Portugal

**Keywords:** Gynaecologic neoplasms, Hereditary cancer syndromes, Neoplasms by histological type, Diagnostic imaging, Practice guideline

## Abstract

**Abstract:**

Hereditary syndromes are responsible for 10 % of gynaecologic cancers, among which hereditary breast-ovarian cancer and hereditary non-polyposis colon cancer syndromes, known as HBOC and Lynch syndromes respectively, present the highest relative risk. The latter predisposes to endometrial cancer and both contribute to ovarian cancer. Cowden syndrome-related endometrial cancer and the increased risk of ovarian, uterine and cervical cancers associated with Peutz-Jeghers syndrome, are also demonstrated, while Li-Fraumeni syndrome patients are prone to develop ovarian and endometrial cancers. Despite these syndromes’ susceptibility to gynaecologic cancers being consensual, it is still not clear whether these tumours have any epidemiologic, clinical, pathologic or imaging specific features that could allow any of the intervening physicians to raise suspicion of a hereditary syndrome in patients without known genetic risk. Moreover, controversy exists regarding both screening and surveillance schemes. Our literature review provides an updated perspective on the evidence-based specific features of tumours related to each of these syndromes as well as on the most accepted screening and surveillance guidelines. In addition, some illustrative cases are presented.

***Teaching Points*:**

• *HBOC syndrome is mainly associated with ovarian HGSC, which arises in fallopian fimbriae.*

• *LS-related endometrial tumours show histological diversity and predilection for lower uterine segment.*

• *LS and CS-related ovarian cancers are mostly of non-serous type, usually endometrioid.*

• *Ovarian SCTAT and cervical adenoma malignum are strongly associated with PJS.*

• *Unfortunately, hereditary gynaecologic cancers do not seem to have distinctive imaging features.*

## Introduction

Recently, there have been significant advances in the knowledge of female genital tract malignancies related to hereditary cancer susceptibility syndromes. According to the American Society of Clinical Oncology, hereditary syndromes are responsible for about 10 % of gynaecologic cancers [1].

One striking example is the discovery of the association between germline mutations in breast cancer (BRCA) 1 and 2 genes and ovarian cancers in hereditary breast-ovarian cancer (HBOC) syndrome. Another one is the role of germline mutations in DNA mismatch repair (MMR) genes in endometrial and ovarian carcinogenesis related to hereditary non-polyposis colon cancer, also known as Lynch syndrome (LS).

Increased risk of endometrial cancer caused by mutation in the phosphatase and tensin homolog (PTEN) gene in Cowden syndrome (CS) is also demonstrated, as well as ovarian, uterine and cervical cancers related to Peutz-Jeghers syndrome (PJS), due to liver kinase b1 (LKB1/STK11) gene mutation. Ovarian and endometrial cancers also occur excessively in patients with Li-Fraumeni syndrome (LFS), although the understanding of the contribution of this inherited germline mutation in p53 is less established.

Despite the clear evidence of these inherited disorders’ susceptibility to gynaecologic cancers, it is still not generically clear whether these tumours have any epidemiologic, clinical, pathologic or imaging specific features that could allow any of the intervening physicians to raise suspicion of a hereditary syndrome in patients without known genetic risk. Moreover, their screening and surveillance schemes remain controversial.

Our literature review provides an updated perspective on the evidence-based specific features of tumours related to each of these syndromes, as well as on the most accepted screening and surveillance guidelines. In addition, some illustrative cases are presented.

## Hereditary breast-ovarian cancer syndrome

Ovarian cancer is the most lethal gynaecologic cancer, 70 % being detected with advanced disease and therefore having poor prognosis, with a 5-year survival rate of only 15 to 25 % for stage IV [2, 3].

90 % have epithelial origin [4], hereditary ones accounting for at least 10 % of cases [2, 5, 6] and the majority being due to mutations in BRCA1 gene [5, 7]. Lifetime risk for ovarian cancers is 40–66 % and 10–20 % in BRCA1 and BRCA2 germline mutation carriers, respectively [8–11], in contrast to 1.8 % in the general population [12].

BRCA1 locus on chromosome 17q and BRCA2 on chromosome 13q both function as tumour suppressor genes [13–15]. These mutations, also associated with increased risk of breast cancer, are both on the basis of HBOC and site-specific ovarian cancer syndromes. Ethnic background significantly influences the mutation rates, which are particularly high among Ashkenazi Jews compared to other populations [16, 17].

Mean age of presentation of ovarian cancer in HBOC syndrome is 51–53 years, about 10 years earlier than in non-BRCA mutations carriers [5, 12].

Concerning its pathology, the great majority are high-grade serous carcinomas (HGSC) of papillary type, diagnosed at advanced stage [2, 7, 12, 18]. Despite this, survival of ovarian cancer seems to be surprisingly better in these women than in sporadic ones [7], for unknown reasons.

**Fig. 1:**
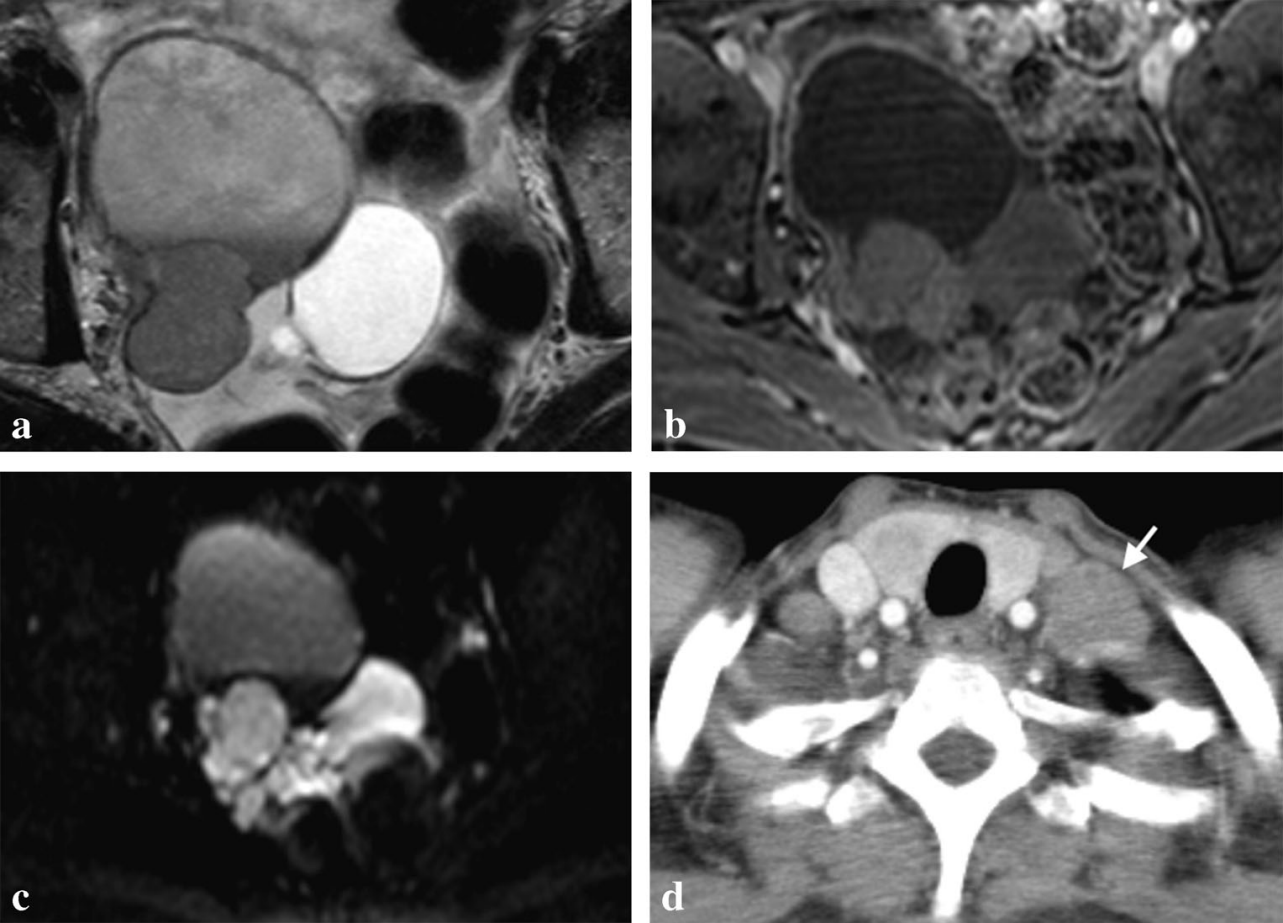
**a**-**d**. High-grade serous carcinoma of the right ovary in a 56-year-old BRCA1-mutation carrier woman. Magnetic resonance imaging (MRI) scan with T2-weighted (**a**), post-gadolinium fat-suppressed (FS) T1-weighted (**b**) and diffusion-weighted (**c**) images, showing a complex cystic-solid multilobulated right adnexal mass with restricted diffusion on b600 image (**c**), with pathologically proved capsule rupture, but without metastases. Contrast-enhanced computed tomography (CECT) obtained 1 year later due to a left supraclavicular palpable painful mass confirms the presence of a lymph node metastasis (*arrow* in **d**)

**Fig. 2:**
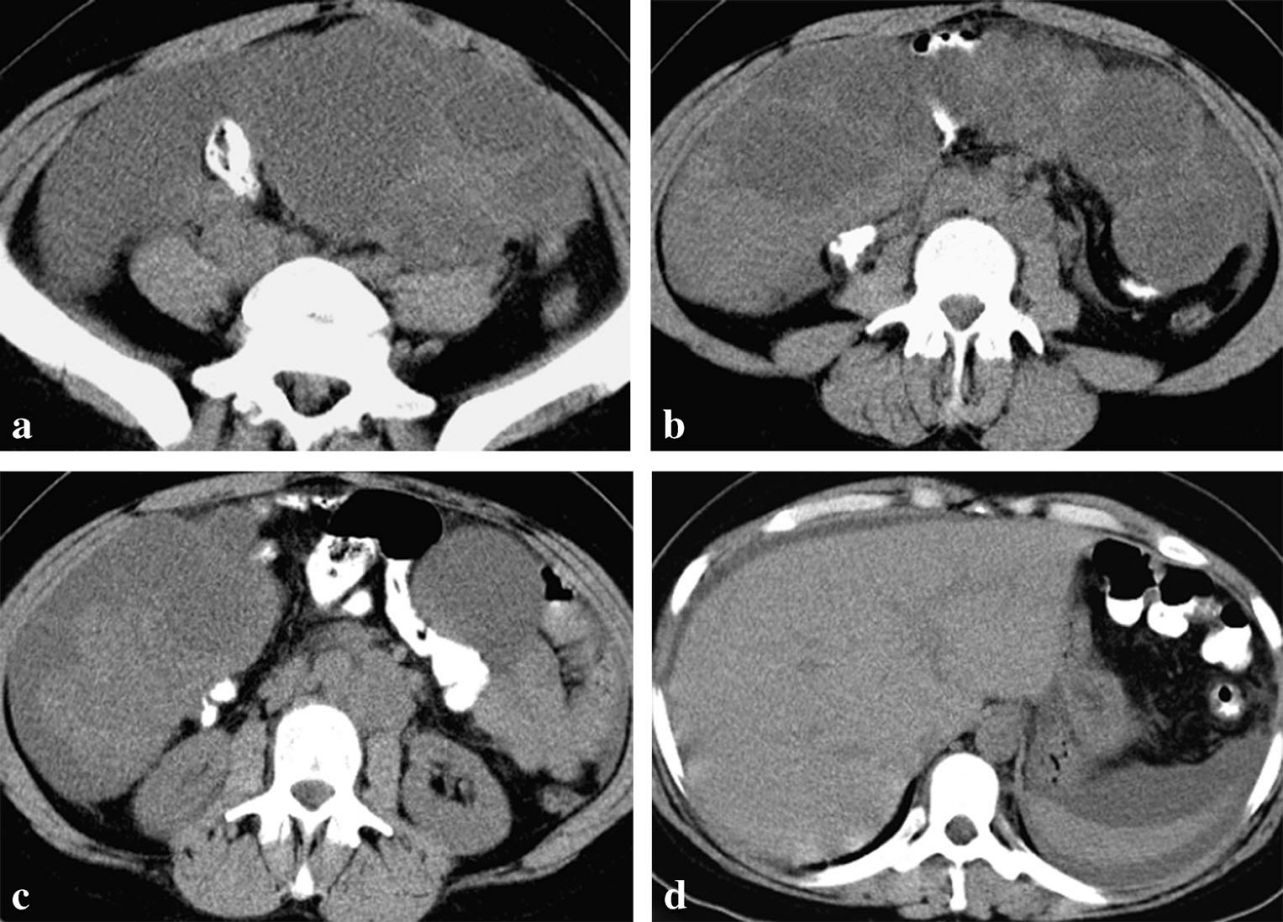
**a**-**d**. Bilateral undifferentiated ovarian carcinomas in a 34-year-old BRCA1-mutation carrier woman. CT scan shows a large multiloculated cystic left adnexal mass with thick septa (**a**). A right adnexal mass, similar to that one described in image **a**, but with a more prominent solid component, was also present (**b**). Left para-aortic lymph node metastases are also present (**c**). Moderate ascites is evident in all images but there was no pleural effusion (**d**). Pathologic examination ruled out ovarian capsule and tubal invasion

**Fig. 3:**
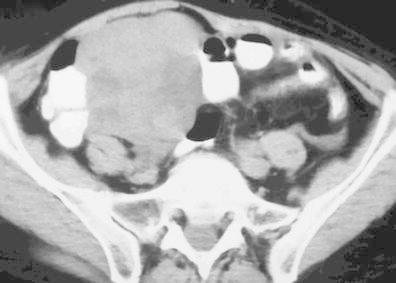
Undifferentiated ovarian carcinoma in a 59-year-old BRCA2-mutation carrier woman. CT scan demonstrates a large multilobulated complex cystic-solid right adnexal mass. There were neither ascites nor enlarged lymph nodes

Both cases of BRCA1 mutation-related ovarian cancer presented (Figs. 1 and 2) were high-grade tumours with no tubal involvement, diagnosed at younger ages than is typical for the general population, which metastasized to lymph nodes. On the other hand, a localized ovarian tumour in a BRCA2 mutation carrier is presented in Fig. 3.

A curious detail regarding the origin of most ovarian HGSC in patients with HBOC, suggested by recent studies of prophylactically removed ovaries, is that instead of arising primarily from the ovary as originally assumed, these tumours actually seem to arise from the fallopian tube fimbriae and are characterized by p53 signature mutations, typical of tubal intraepithelial carcinomas. In addition, these studies failed to identify reproducible precursor lesions in the ovaries [2, 12]. For these reasons, some experts consider ovarian and fallopian tube cancer in BRCA-mutations carriers to be more properly termed as adnexal carcinomas [19]. However, interpretation of p53 signatures, defined as p53 positivity by immunohistochemical staining in at least 12 secretory cells with a low proliferative index, must be done cautiously, as it does not mean a neoplastic lesion is present and is a quite common occurrence in fallopian tubes, regardless of BRCA status [12].

Another cancer type, in whose progression the loss of BRCA1 function with concurrent deletion of p53 may be an important step, is uterine leiomyosarcoma, a rare gynaecologic malignancies with a low survival rate [20].

As genetic testing for BRCA genes is not cost-effective for the general population, thus far the selection of candidates still relies on family history [1]. Once a genetic risk is confirmed, management is not consensual, but usually includes serial serum determinations of multiple tumour markers together with transvaginal ultrasound, another option being prophylactic oophorectomy after planned childbearing is completed. Although a protective effect of oral contraceptives against ovarian cancer is nearly proven, their routine prophylactic use is no longer recommended due to the possible increased relative risk of breast cancer in patients with HBOC syndrome [5].

## Lynch syndrome

Lynch syndrome (LS), caused by an autosomal-dominant hereditary germline mutation in one of the MMR genes—MSH2, MLH1 and MSH6, in decreasing order [21]—predisposes to early onset of multiple cancer types, including colon, endometrial and ovarian ones, sometimes with synchronous presentation [22, 23]. MMR maintain genomic stability by correcting mismatches generated during DNA replication, their malfunction promoting cancer due to microsatellite instability [24]. However, microsatellite instability is also present in 15–20 % of corresponding sporadic cancers, usually due to MLH1 methylation [25].

Traditionally associated with colorectal cancer, it is nowadays consensual that women with LS are at equal or even higher risk for development of gynaecologic malignancy [26–29], as their sentinel cancer in more than half of cases [30, 31]. Their lifetime cumulative risk of endometrial cancer is 40–60 % [26, 28, 29, 31] and that of ovarian cancer is 10–12 % [18, 21, 27, 32–34], appearing to be particularly high for MSH2-mutation carriers [35–37] and accounting for 2 % of all ovarian cancers [34].

Although it has long been thought that the average age of onset of LS-related endometrial cancer is 45–50 years, in contrast to 65 years in sporadic cases [5, 38, 39], other studies have suggested that a cutoff age of 50 years old for screening could lead to 25–60 % of LS missing cases [40, 41], therefore a cutoff age of 60 has already been advocated [22]. Regardless of this controversy, a diagnosis of endometrial cancer at a young age should raise suspicion for LS, especially if the most typical constitutional factor of sporadic endometrial cancer—obesity—is absent and family history is positive [42, 43]. Mean age for developing ovarian cancer is 40–48 years among patients with LS [5, 36, 39].

While sporadic endometrial cancers that have microsatellite instability are almost exclusively of endometrioid type, usually well to moderately differentiated, those related to LS tend to be histologically more diverse also occurring non-endometrioid carcinomas, including serous, clear cell and undifferentiated ones, although the majority is still of endometrioid type [38, 43]. LS-related endometrial cancers also show a predilection for the lower uterine segment, with up to one-third of these tumours arising in this location [44–46].

Although certain distinctive microscopic features of endometrioid carcinomas, like poor differentiation, tumour heterogeneity and increased tumour-infiltrating lymphocytes, have been shown to be suggestive of the presence of high levels of microsatellite instability [40, 47, 48], there are conflicting data regarding their utility [46].

Contrary to ovarian cancers of the general population or that are HBOC syndrome-related, those related to LS are mostly of the non-serous type, including endometrioid, clear cell and undifferentiated carcinomas [36, 49]. Endometrioid is most commonly associated with LS [36] and the second most frequent histological subtype in the general population [50], the majority being well to moderately differentiated and pursuing favourable clinical outcomes [36, 51]. There may be synchronous endometrial thickening, representing either hyperplasia or carcinoma [52]. Among ovarian carcinomas associated with MMR defects, including LS-related ones, clear cell subtype represents the majority [35].

Prognostic impact of MMR status is not clear for either endometrial or ovarian carcinomas [12].

**Fig. 4:**
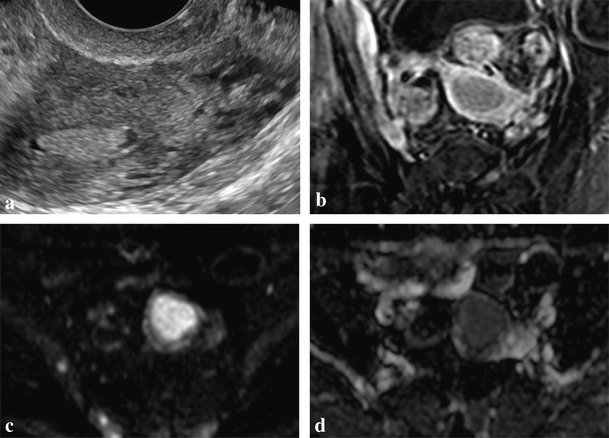
**a**–**d**. Mixed serous-endometrioid carcinoma of the uterus in a 66-year-old woman with Lynch syndrome. Ultrasound (**a**) shows a solid polypoid mass inside the uterine cavity and a small hydrometra. MRI scan demonstrates a solid well-circumscribed endometrial tumour, hypoenhancing compared with myometrium on post-gadolinium FS T1-weighted image (**b**). Diffusion restriction on the b1000 image (**c**) with a low apparent diffusion coefficient (ADC) value (**d**) is also evident, consistent with its malignant nature. The tumour was confined to the corpus uteri with no myometrial invasion

**Fig. 5:**
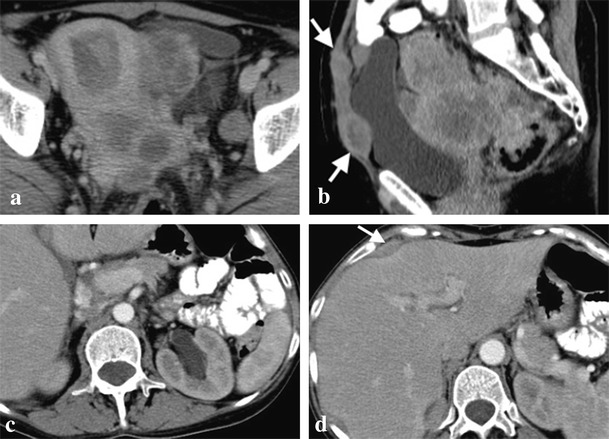
**a**–**d**. Poorly differentiated endometrioid carcinomas of the left ovary and of the endometrium in a 56-year-old woman with Lynch syndrome. CECT shows a large complex cystic-solid multiloculated left adnexal mass with no cleavage plane from the uterine corpus (**a**) in concordance with invasion of the myometrium, as pathologically demonstrated. The same image depicts the endometrial tumour, seen as a polypoid solid lesion within the uterine cavity surrounded by fluid, proved to be confined to the inner half of the endometrium. A CECT scan obtained 2 months after hysterectomy with bilateral adnexectomy without pelvic lymphadenectomy demonstrates in the sagittal plane (**b**) a large pelvic mass arising from the vaginal cuff consistent with tumour recurrence, as well as two abdominal wall implants (*arrows*). Left hydronephrosis secondary to pelvic wall invasion (**c**) and a hepatic subcapsular implant (**d**) are also evident in the same study

LS cases illustrated in Figs. 4 and 5 correspond to patients whose sentinel cancers were gynaecologic, the latter with synchronous endometrial and ovarian tumours.

Current gynaecologic cancer screening guidelines for women with LS, which include annual endometrial sampling and transvaginal ultrasound beginning at 30–35 years, are not considered to be effective, but are still a reasonable option [12, 53].

## Cowden syndrome

Cowden syndrome (CS), part of a broader category termed PTEN hamartoma tumour syndrome, is characterized by a mutation in the PTEN tumour-suppressor gene, which leads to uncontrolled cell division and the formation of hamartomatous neoplasms and certain cancers, representing an increased lifetime risk of endometrial carcinomas of 13–19 % [54].

**Fig. 6:**
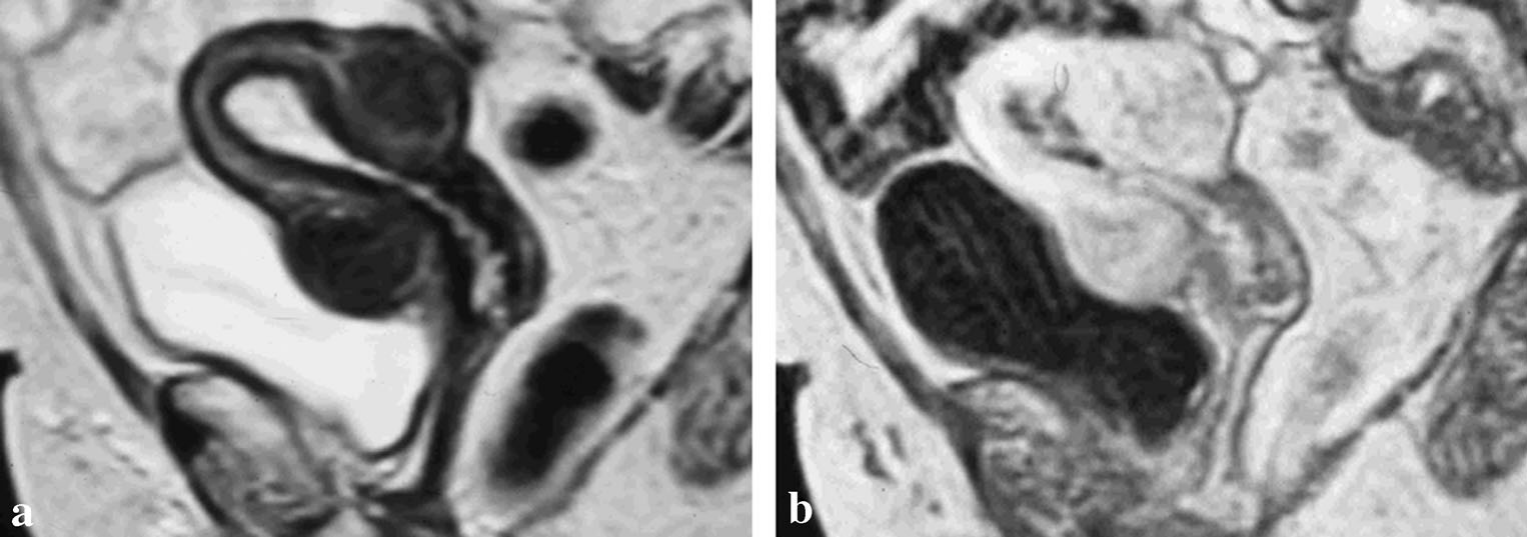
**a**, **b**. High-grade endometrioid carcinoma within an endometrial hyperplastic polyp in a 62-year-old woman with Cowden syndrome. T2-weighted image (**a**) shows distension of the uterine cavity due to a polyp, more evident on post-gadolinium FS T1-weighted image (**b**), which is hypoenhancing compared with myometrium. Normal uterine zonal anatomy is preserved. Note the bosselated and smooth outer contour of the uterus due to leiomyomas

Histologically, reported cases of CS-related endometrial carcinomas [54–57] and the one illustrated in Fig. 6 are of endometrioid type.

Although still not validated, the adoption of the screening guidelines for LS, including annual endometrial biopsies beginning at age 30 to 35, or 5 years before the earliest family diagnosis of endometrial cancer and annual ultrasound examination with biopsy of suspicious areas for postmenopausal women, has already been proposed [54]. Formal evaluation for CS when endometrial cancer is diagnosed in adolescence has also been suggested [55].

## Li-fraumeni syndrome

Li-fraumeni syndrome (LFS) is an extremely rare autosomal-dominant hereditary disorder characterized by a germline mutation in the tumour-suppression gene p53, which leads to an estimated 50-fold risk over the general population of developing several types of cancer [58], more than half occurring before age 30 [59]. Although endometrial and ovarian cancers have been found to occur excessively in at least some families who have met criteria for LFS, their link to the syndrome is not definitely established, occurring at a much lower rate than other cancer types, like breast cancer [60].

Actually, the fact that distal fallopian tubes of women with LFS are exquisitely prone to developing p53 signatures, identical to those described in BRCA1/2-mutations carriers and general population, does not mean an association with ovarian cancer, as at least one more genotoxic event is needed to produce the malignant phenotype [61].

There are no studies specifically examining the relation between LFS and endometrial cancer.

## Peutz-jeghers syndrome

Peutz-jeghers syndrome (PJS) is a rare autosomal dominant disease due to mutations in the tumour-suppressor gene STK11 [62], which predisposes not only to hamartomatous gastrointestinal polyps and mucocutaneous pigmentation, but also to numerous malignancies, including gynaecologic ones, the latter having a reported relative risk of 27.7 % in comparison to the general population [63].

Risk of ovarian, cervical and uterine cancers associated with PJS is 18–21 %, 10 % and 9 %, respectively [64].

PJS-related gynaecological cancers are of some characteristic histological types, particularly the sex cord tumours with annular tubules (SCTAT) of the ovary [63, 65–67], 36 % occurring in association with this syndrome [65], although with lower risk of malignant transformation than in the general population [63]. This distinctive ovarian neoplasm, whose predominant component has morphologic features intermediate between those of granulosa cell and Sertoli cell tumours, may produce both oestrogen and progesterone [68]. This histological type is followed by Sertoli cell, mucinous, serous and mature teratoma [67].

There is also evidence that patients with PJS are prone to develop endometrial adenocarcinomas [69, 70], especially highly invasive ones [70].

Among cervical tumours, there is an important association with minimal deviation adenocarcinoma [65, 66, 71], known as adenoma malignum, with 10 % of all cases occurring in PJS patients [66]. This is a well-differentiated mucinous adenocarcinoma with highly aggressive behaviour, despite its deceptively benign appearance and very scarce cytological features of malignancy within the tumour [71].

Gynaecologic cancer screening surveillance recommendations for patients with PJS include annual Papanicolaou test by age 18 and annual pelvic examination and ultrasound by age 20 [72], which should also target the potential malignant change of ovarian SCTAT [63].

## Conclusions

Regardless of some genetic specifications, the following particularities concerning each of the syndromes discussed above are consensual nowadays:

- HBOC syndrome is mainly associated with ovarian HGSC, which seem to arise in the fallopian fimbriae and have better prognosis than sporadic cancers.
- LS predisposes to endometrial cancer, at a lower rate than ovarian cancers. Endometrial cancers show a predilection for the lower uterine segment and tend to be histologically more diverse in contrast to their sporadic counterparts, including non-endometrioid carcinomas. Both LS and CS-related ovarian cancers are mostly of non-serous type, usually endometrioid.
- LFS only slightly increases the risk of endometrial and ovarian cancers.
- PJS increases the risk of ovarian, cervical and uterine cancers, in decreasing order, ovarian SCTAT and cervical adenoma malignum being strongly associated.
- Unfortunately, hereditary gynaecologic cancers do not seem to have any imaging characteristics that might be reliably used to distinguish them from sporadic cancers.
- In order to ensure early identification of high-risk patients, every time a gynaecologic malignancy is diagnosed under the expected age in the general population, a careful anamnesis including familial history of cancer and genetic confirmation when indicated is required.
- Screening and surveillance schemes usually consist of an annual pelvic examination with endometrial sampling and ultrasound beginning in young adulthood.
